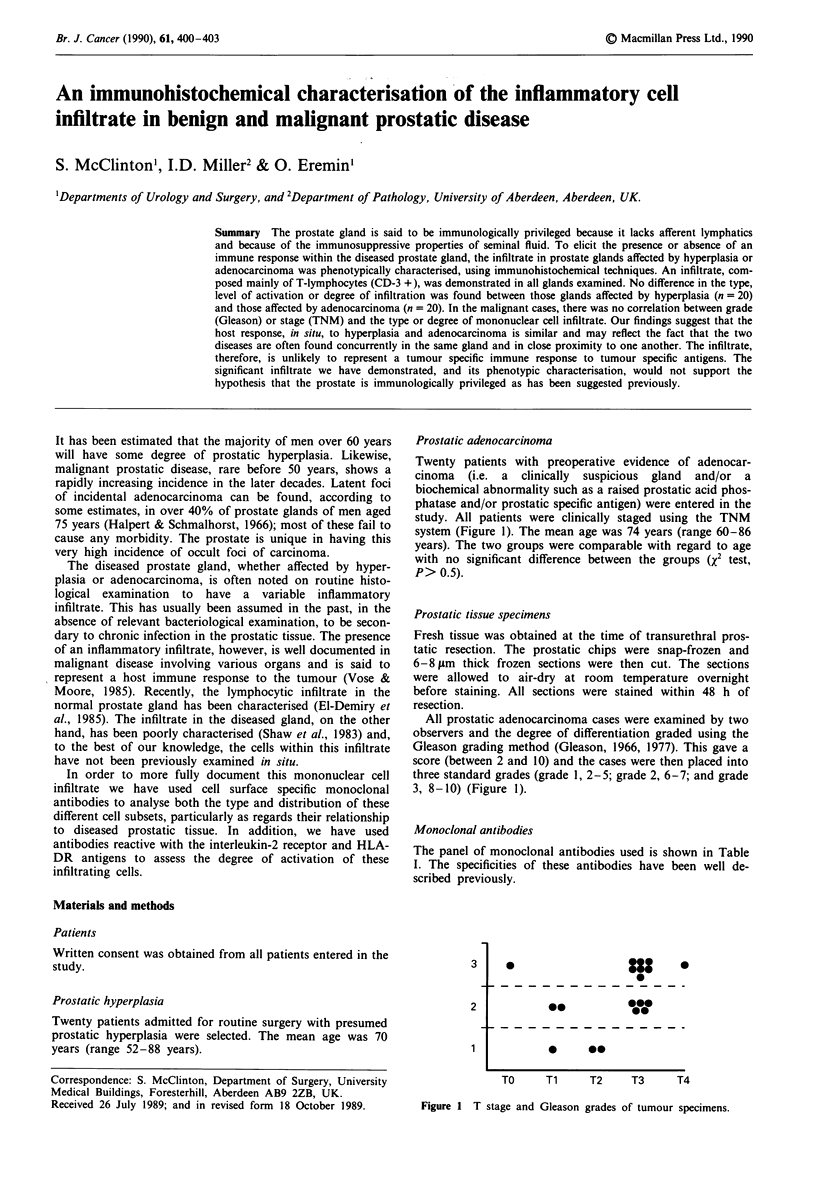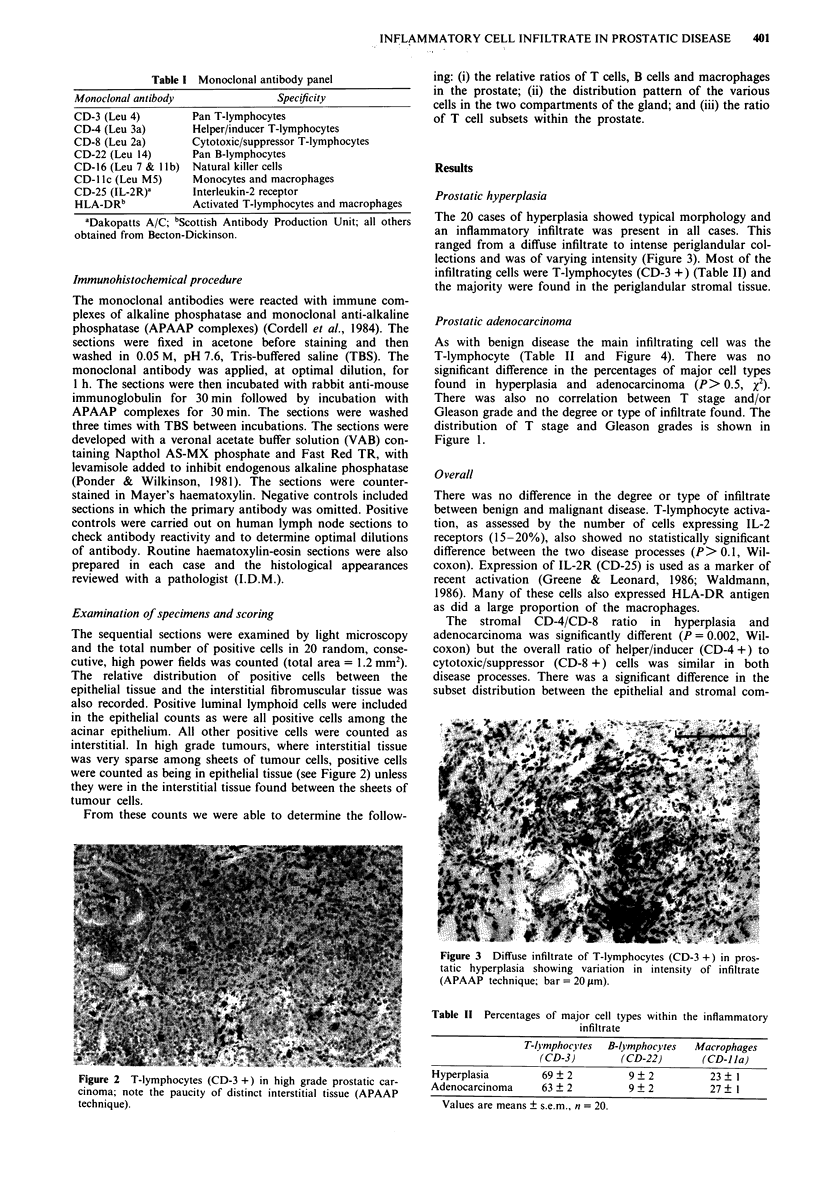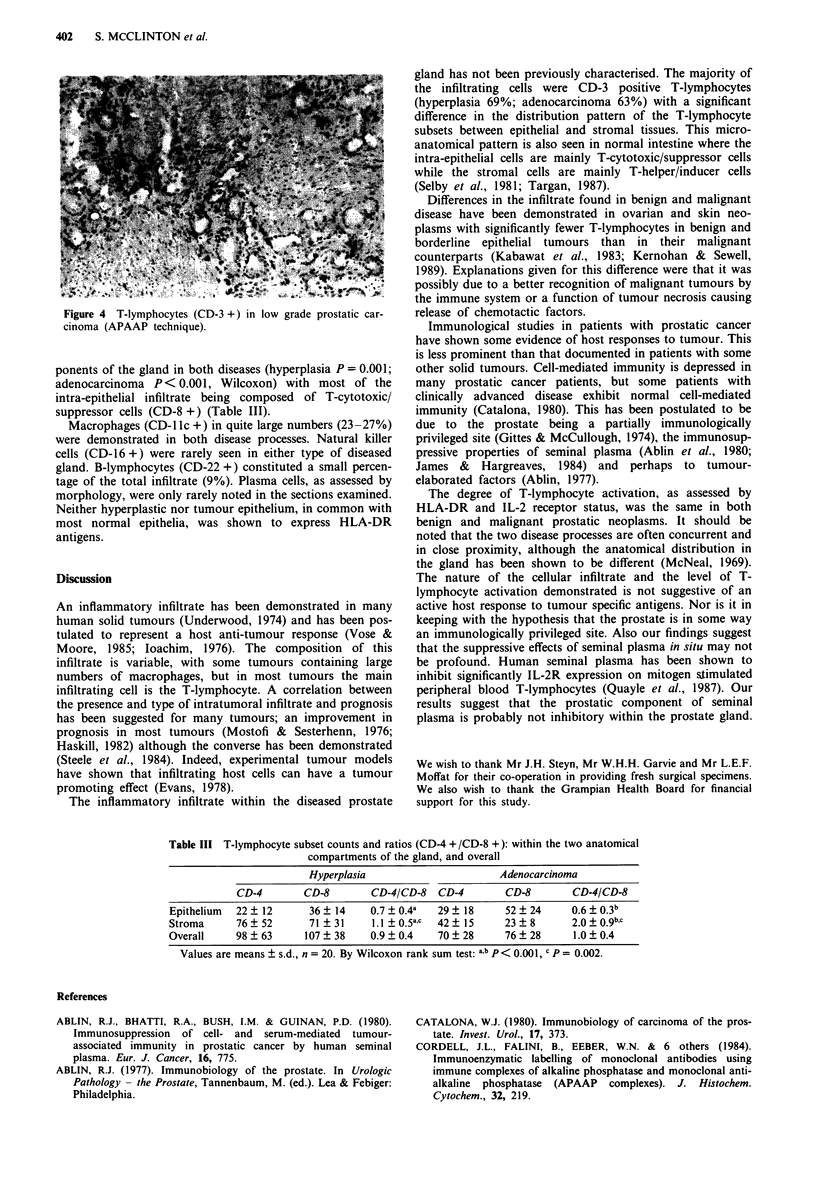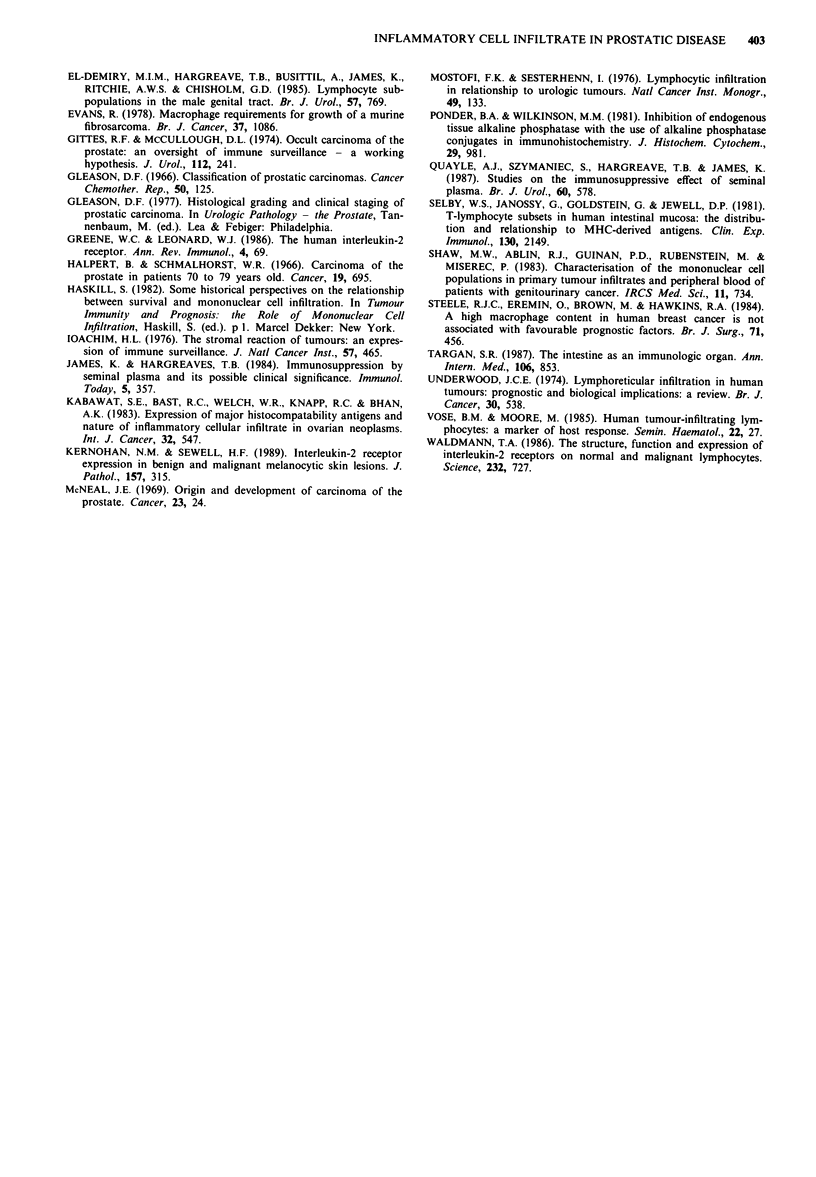# An immunohistochemical characterisation of the inflammatory cell infiltrate in benign and malignant prostatic disease.

**DOI:** 10.1038/bjc.1990.87

**Published:** 1990-03

**Authors:** S. McClinton, I. D. Miller, O. Eremin

**Affiliations:** Department of Urology, University of Aberdeen, UK.

## Abstract

**Images:**


 © Macmillan Press Ltd., 1990

### An immunohistochemical characterisation of the inflammatory cell infiltrate in benign and malignant prostatic disease

S. McClinton[1], I.D. Miller[2] & O. Eremin[1]

[1]*Departments of Urology and Surgery, and* [2]*Department of Pathology, University of Aberdeen, Aberdeen, UK.*

**Summary** The prostate gland is said to be immunologically privileged because it lacks afferent lymphatics and because of the immunosuppressive properties of seminal fluid. To elicit the presence or absence of an immune response within the diseased prostate gland, the infiltrate in prostate glands affected by hyperplasia or adenocarcinoma was phenotypically characterised, using immunohistochemical techniques. An infiltrate, composed mainly of T-lymphocytes (CD-3 +), was demonstrated in all glands examined. No difference in the type, level of activation or degree of infiltration was found between those glands affected by hyperplasia ($n = 20$) and those affected by adenocarcinoma ($n = 20$). In the malignant cases, there was no correlation between grade (Gleason) or stage (TNM) and the type or degree of mononuclear cell infiltrate. Our findings suggest that the host response, *in situ*, to hyperplasia and adenocarcinoma is similar and may reflect the fact that the two diseases are often found concurrently in the same gland and in close proximity to one another. The infiltrate, therefore, is unlikely to represent a tumour specific immune response to tumour specific antigens. The significant infiltrate we have demonstrated, and its phenotypic characterisation, would not support the hypothesis that the prostate is immunologically privileged as has been suggested previously.

It has been estimated that the majority of men over 60 years will have some degree of prostatic hyperplasia. Likewise, malignant prostatic disease, rare before 50 years, shows a rapidly increasing incidence in the later decades. Latent foci of incidental adenocarcinoma can be found, according to some estimates, in over 40% of prostate glands of men aged 75 years (Halpert & Schmalhorst, 1966); most of these fail to cause any morbidity. The prostate is unique in having this very high incidence of occult foci of carcinoma.

The diseased prostate gland, whether affected by hyperplasia or adenocarcinoma, is often noted on routine histological examination to have a variable inflammatory infiltrate. This has usually been assumed in the past, in the absence of relevant bacteriological examination, to be secondary to chronic infection in the prostatic tissue. The presence of an inflammatory infiltrate, however, is well documented in malignant disease involving various organs and is said to represent a host immune response to the tumour (Vose & Moore, 1985). Recently, the lymphocytic infiltrate in the normal prostate gland has been characterised (El-Demiry *et al.*, 1985). The infiltrate in the diseased gland, on the other hand, has been poorly characterised (Shaw *et al.*, 1983) and, to the best of our knowledge, the cells within this infiltrate have not been previously examined *in situ*.

In order to more fully document this mononuclear cell infiltrate we have used cell surface specific monoclonal antibodies to analyse both the type and distribution of these different cell subsets, particularly as regards their relationship to diseased prostatic tissue. In addition, we have used antibodies reactive with the interleukin-2 receptor and HLA-DR antigens to assess the degree of activation of these infiltrating cells.

### Materials and methods

*Patients*

Written consent was obtained from all patients entered in the study.

*Prostatic hyperplasia*

Twenty patients admitted for routine surgery with presumed prostatic hyperplasia were selected. The mean age was 70 years (range 52–88 years).

Correspondence: S. McClinton, Department of Surgery, University Medical Buildings, Foresterhill, Aberdeen AB9 2ZB, UK.
Received 26 July 1989; and in revised form 18 October 1989.

*Prostatic adenocarcinoma*

Twenty patients with preoperative evidence of adenocarcinoma (i.e. a clinically suspicious gland and/or a biochemical abnormality such as a raised prostatic acid phosphatase and/or prostatic specific antigen) were entered in the study. All patients were clinically staged using the TNM system (Figure 1). The mean age was 74 years (range 60–86 years). The two groups were comparable with regard to age with no significant difference between the groups ($\chi^2$ test, $P > 0.5$).

*Prostatic tissue specimens*

Fresh tissue was obtained at the time of transurethral prostatic resection. The prostatic chips were snap-frozen and 6–8 μm thick frozen sections were then cut. The sections were allowed to air-dry at room temperature overnight before staining. All sections were stained within 48 h of resection.

All prostatic adenocarcinoma cases were examined by two observers and the degree of differentiation graded using the Gleason grading method (Gleason, 1966, 1977). This gave a score (between 2 and 10) and the cases were then placed into three standard grades (grade 1, 2–5; grade 2, 6–7; and grade 3, 8–10) (Figure 1).

*Monoclonal antibodies*

The panel of monoclonal antibodies used is shown in Table I. The specificities of these antibodies have been well described previously.

**Figure 1** T stage and Gleason grades of tumour specimens.

**Table I** Monoclonal antibody panel

| *Monoclonal antibody* | *Specificity* |
|---|---|
| CD-3 (Leu 4) | Pan T-lymphocytes |
| CD-4 (Leu 3a) | Helper/inducer T-lymphocytes |
| CD-8 (Leu 2a) | Cytotoxic/suppressor T-lymphocytes |
| CD-22 (Leu 14) | Pan B-lymphocytes |
| CD-16 (Leu 7 & 11b) | Natural killer cells |
| CD-11c (Leu M5) | Monocytes and macrophages |
| CD-25 (IL-2R)[a] | Interleukin-2 receptor |
| HLA-DR[b] | Activated T-lymphocytes and macrophages |

[a]Dakopatts A/C; [b]Scottish Antibody Production Unit; all others obtained from Becton-Dickinson.

*Immunohistochemical procedure*

The monoclonal antibodies were reacted with immune complexes of alkaline phosphatase and monoclonal anti-alkaline phosphatase (APAAP complexes) (Cordell *et al.*, 1984). The sections were fixed in acetone before staining and then washed in 0.05 M, pH 7.6, Tris-buffered saline (TBS). The monoclonal antibody was applied, at optimal dilution, for 1 h. The sections were then incubated with rabbit anti-mouse immunoglobulin for 30 min followed by incubation with APAAP complexes for 30 min. The sections were washed three times with TBS between incubations. The sections were developed with a veronal acetate buffer solution (VAB) containing Napthol AS-MX phosphate and Fast Red TR, with levamisole added to inhibit endogenous alkaline phosphatase (Ponder & Wilkinson, 1981). The sections were counterstained in Mayer's haematoxylin. Negative controls included sections in which the primary antibody was omitted. Positive controls were carried out on human lymph node sections to check antibody reactivity and to determine optimal dilutions of antibody. Routine haematoxylin-eosin sections were also prepared in each case and the histological appearances reviewed with a pathologist (I.D.M.).

*Examination of specimens and scoring*

The sequential sections were examined by light microscopy and the total number of positive cells in 20 random, consecutive, high power fields was counted (total area = 1.2 $mm^2$). The relative distribution of positive cells between the epithelial tissue and the interstitial fibromuscular tissue was also recorded. Positive luminal lymphoid cells were included in the epithelial counts as were all positive cells among the acinar epithelium. All other positive cells were counted as interstitial. In high grade tumours, where interstitial tissue was very sparse among sheets of tumour cells, positive cells were counted as being in epithelial tissue (see Figure 2) unless they were in the interstitial tissue found between the sheets of tumour cells.

From these counts we were able to determine the following: (i) the relative ratios of T cells, B cells and macrophages in the prostate; (ii) the distribution pattern of the various cells in the two compartments of the gland; and (iii) the ratio of T cell subsets within the prostate.

**Figure 2** T-lymphocytes (CD-3 +) in high grade prostatic carcinoma; note the paucity of distinct interstitial tissue (APAAP technique).

### Results

*Prostatic hyperplasia*

The 20 cases of hyperplasia showed typical morphology and an inflammatory infiltrate was present in all cases. This ranged from a diffuse infiltrate to intense periglandular collections and was of varying intensity (Figure 3). Most of the infiltrating cells were T-lymphocytes (CD-3 +) (Table II) and the majority were found in the periglandular stromal tissue.

*Prostatic adenocarcinoma*

As with benign disease the main infiltrating cell was the T-lymphocyte (Table II and Figure 4). There was no significant difference in the percentages of major cell types found in hyperplasia and adenocarcinoma ($P > 0.5$, $\chi^2$). There was also no correlation between T stage and/or Gleason grade and the degree or type of infiltrate found. The distribution of T stage and Gleason grades is shown in Figure 1.

*Overall*

There was no difference in the degree or type of infiltrate between benign and malignant disease. T-lymphocyte activation, as assessed by the number of cells expressing IL-2 receptors (15–20%), also showed no statistically significant difference between the two disease processes ($P > 0.1$, Wilcoxon). Expression of IL-2R (CD-25) is used as a marker of recent activation (Greene & Leonard, 1986; Waldmann, 1986). Many of these cells also expressed HLA-DR antigen as did a large proportion of the macrophages.

The stromal CD-4/CD-8 ratio in hyperplasia and adenocarcinoma was significantly different ($P = 0.002$, Wilcoxon) but the overall ratio of helper/inducer (CD-4 +) to cytotoxic/suppressor (CD-8 +) cells was similar in both disease processes. There was a significant difference in the subset distribution between the epithelial and stromal com-

**Figure 3** Diffuse infiltrate of T-lymphocytes (CD-3 +) in prostatic hyperplasia showing variation in intensity of infiltrate (APAAP technique; bar = 20 μm).

**Table II** Percentages of major cell types within the inflammatory infiltrate

| | *T-lymphocytes (CD-3)* | *B-lymphocytes (CD-22)* | *Macrophages (CD-11a)* |
|---|---|---|---|
| Hyperplasia | 69 ± 2 | 9 ± 2 | 23 ± 1 |
| Adenocarcinoma | 63 ± 2 | 9 ± 2 | 27 ± 1 |

Values are means ± s.e.m., $n = 20$.

**Figure 4** T-lymphocytes (CD-3 +) in low grade prostatic carcinoma (APAAP technique).

ponents of the gland in both diseases (hyperplasia $P = 0.001$; adenocarcinoma $P < 0.001$, Wilcoxon) with most of the intra-epithelial infiltrate being composed of T-cytotoxic/suppressor cells (CD-8 +) (Table III).

Macrophages (CD-11c +) in quite large numbers (23–27%) were demonstrated in both disease processes. Natural killer cells (CD-16 +) were rarely seen in either type of diseased gland. B-lymphocytes (CD-22 +) constituted a small percentage of the total infiltrate (9%). Plasma cells, as assessed by morphology, were only rarely noted in the sections examined. Neither hyperplastic nor tumour epithelium, in common with most normal epithelia, was shown to express HLA-DR antigens.

### Discussion

An inflammatory infiltrate has been demonstrated in many human solid tumours (Underwood, 1974) and has been postulated to represent a host anti-tumour response (Vose & Moore, 1985; Ioachim, 1976). The composition of this infiltrate is variable, with some tumours containing large numbers of macrophages, but in most tumours the main infiltrating cell is the T-lymphocyte. A correlation between the presence and type of intratumoral infiltrate and prognosis has been suggested for many tumours; an improvement in prognosis in most tumours (Mostofi & Sesterhenn, 1976; Haskill, 1982) although the converse has been demonstrated (Steele *et al.*, 1984). Indeed, experimental tumour models have shown that infiltrating host cells can have a tumour promoting effect (Evans, 1978).

The inflammatory infiltrate within the diseased prostate gland has not been previously characterised. The majority of the infiltrating cells were CD-3 positive T-lymphocytes (hyperplasia 69%; adenocarcinoma 63%) with a significant difference in the distribution pattern of the T-lymphocyte subsets between epithelial and stromal tissues. This micro-anatomical pattern is also seen in normal intestine where the intra-epithelial cells are mainly T-cytotoxic/suppressor cells while the stromal cells are mainly T-helper/inducer cells (Selby *et al.*, 1981; Targan, 1987).

Differences in the infiltrate found in benign and malignant disease have been demonstrated in ovarian and skin neoplasms with significantly fewer T-lymphocytes in benign and borderline epithelial tumours than in their malignant counterparts (Kabawat *et al.*, 1983; Kernohan & Sewell, 1989). Explanations given for this difference were that it was possibly due to a better recognition of malignant tumours by the immune system or a function of tumour necrosis causing release of chemotactic factors.

Immunological studies in patients with prostatic cancer have shown some evidence of host responses to tumour. This is less prominent than that documented in patients with some other solid tumours. Cell-mediated immunity is depressed in many prostatic cancer patients, but some patients with clinically advanced disease exhibit normal cell-mediated immunity (Catalona, 1980). This has been postulated to be due to the prostate being a partially immunologically privileged site (Gittes & McCullough, 1974), the immunosuppressive properties of seminal plasma (Ablin *et al.*, 1980; James & Hargreaves, 1984) and perhaps to tumour-elaborated factors (Ablin, 1977).

The degree of T-lymphocyte activation, as assessed by HLA-DR and IL-2 receptor status, was the same in both benign and malignant prostatic neoplasms. It should be noted that the two disease processes are often concurrent and in close proximity, although the anatomical distribution in the gland has been shown to be different (McNeal, 1969). The nature of the cellular infiltrate and the level of T-lymphocyte activation demonstrated is not suggestive of an active host response to tumour specific antigens. Nor is it in keeping with the hypothesis that the prostate is in some way an immunologically privileged site. Also our findings suggest that the suppressive effects of seminal plasma *in situ* may not be profound. Human seminal plasma has been shown to inhibit significantly IL-2R expression on mitogen stimulated peripheral blood T-lymphocytes (Quayle *et al.*, 1987). Our results suggest that the prostatic component of seminal plasma is probably not inhibitory within the prostate gland.

We wish to thank Mr J.H. Steyn, Mr W.H.H. Garvie and Mr L.E.F. Moffat for their co-operation in providing fresh surgical specimens. We also wish to thank the Grampian Health Board for financial support for this study.

**Table III** T-lymphocyte subset counts and ratios (CD-4 +/CD-8 +): within the two anatomical compartments of the gland, and overall

| | *Hyperplasia* | | | *Adenocarcinoma* | | |
|---|---|---|---|---|---|---|
| | *CD-4* | *CD-8* | *CD-4/CD-8* | *CD-4* | *CD-8* | *CD-4/CD-8* |
| Epithelium | 22 ± 12 | 36 ± 14 | $0.7 \pm 0.4^{a}$ | 29 ± 18 | 52 ± 24 | $0.6 \pm 0.3^{b}$ |
| Stroma | 76 ± 52 | 71 ± 31 | $1.1 \pm 0.5^{a,c}$ | 42 ± 15 | 23 ± 8 | $2.0 \pm 0.9^{b,c}$ |
| Overall | 98 ± 63 | 107 ± 38 | 0.9 ± 0.4 | 70 ± 28 | 76 ± 28 | 1.0 ± 0.4 |

Values are means ± s.d., $n = 20$. By Wilcoxon rank sum test: $^{a,b}$ $P < 0.001$, $^{c}$ $P = 0.002$.

### References

ABLIN, R.J., BHATTI, R.A., BUSH, I.M. & GUINAN, P.D. (1980). Immunosuppression of cell- and serum-mediated tumour-associated immunity in prostatic cancer by human seminal plasma. *Eur. J. Cancer*, **16**, 775.

ABLIN, R.J. (1977). Immunobiology of the prostate. In *Urologic Pathology – the Prostate*, Tannenbaum, M. (ed.). Lea & Febiger: Philadelphia.

CATALONA, W.J. (1980). Immunobiology of carcinoma of the prostate. *Invest. Urol.*, **17**, 373.

CORDELL, J.L., FALINI, B., EEBER, W.N. & 6 others (1984). Immunoenzymatic labelling of monoclonal antibodies using immune complexes of alkaline phosphatase and monoclonal anti-alkaline phosphatase (APAAP complexes). *J. Histochem. Cytochem.*, **32**, 219.

EL-DEMIRY, M.I.M., HARGREAVE, T.B., BUSITTIL, A., JAMES, K., RITCHIE, A.W.S. & CHISHOLM, G.D. (1985). Lymphocyte subpopulations in the male genital tract. *Br. J. Urol.*, **57**, 769.

EVANS, R. (1978). Macrophage requirements for growth of a murine fibrosarcoma. *Br. J. Cancer*, **37**, 1086.

GITTES, R.F. & MCCULLOUGH, D.L. (1974). Occult carcinoma of the prostate: an oversight of immune surveillance – a working hypothesis. *J. Urol.*, **112**, 241.

GLEASON, D.F. (1966). Classification of prostatic carcinomas. *Cancer Chemother. Rep.*, **50**, 125.

GLEASON, D.F. (1977). Histological grading and clinical staging of prostatic carcinoma. In *Urologic Pathology – the Prostate*, Tannenbaum, M. (ed.). Lea & Febiger: Philadelphia.

GREENE, W.C. & LEONARD, W.J. (1986). The human interleukin-2 receptor. *Ann. Rev. Immunol.*, **4**, 69.

HALPERT, B. & SCHMALHORST, W.R. (1966). Carcinoma of the prostate in patients 70 to 79 years old. *Cancer*, **19**, 695.

HASKILL, S. (1982). Some historical perspectives on the relationship between survival and mononuclear cell infiltration. In *Tumour Immunity and Prognosis: the Role of Mononuclear Cell Infiltration*, Haskill, S. (ed.). p 1. Marcel Dekker: New York.

IOACHIM, H.L. (1976). The stromal reaction of tumours: an expression of immune surveillance. *J. Natl Cancer Inst.*, **57**, 465.

JAMES, K. & HARGREAVES, T.B. (1984). Immunosuppression by seminal plasma and its possible clinical significance. *Immunol. Today*, **5**, 357.

KABAWAT, S.E., BAST, R.C., WELCH, W.R., KNAPP, R.C. & BHAN, A.K. (1983). Expression of major histocompatability antigens and nature of inflammatory cellular infiltrate in ovarian neoplasms. *Int. J. Cancer*, **32**, 547.

KERNOHAN, N.M. & SEWELL, H.F. (1989). Interleukin-2 receptor expression in benign and malignant melanocytic skin lesions. *J. Pathol.*, **157**, 315.

McNEAL, J.E. (1969). Origin and development of carcinoma of the prostate. *Cancer*, **23**, 24.

MOSTOFI, F.K. & SESTERHENN, I. (1976). Lymphocytic infiltration in relationship to urologic tumours. *Natl Cancer Inst. Monogr.*, **49**, 133.

PONDER, B.A. & WILKINSON, M.M. (1981). Inhibition of endogenous tissue alkaline phosphatase with the use of alkaline phosphatase conjugates in immunohistochemistry. *J. Histochem. Cytochem.*, **29**, 981.

QUAYLE, A.J., SZYMANIEC, S., HARGREAVE, T.B. & JAMES, K. (1987). Studies on the immunosuppressive effect of seminal plasma. *Br. J. Urol.*, **60**, 578.

SELBY, W.S., JANOSSY, G., GOLDSTEIN, G. & JEWELL, D.P. (1981). T-lymphocyte subsets in human intestinal mucosa: the distribution and relationship to MHC-derived antigens. *Clin. Exp. Immunol.*, **130**, 2149.

SHAW, M.W., ABLIN, R.J., GUINAN, P.D., RUBENSTEIN, M. & MISEREC, P. (1983). Characterisation of the mononuclear cell populations in primary tumour infiltrates and peripheral blood of patients with genitourinary cancer. *IRCS Med. Sci.*, **11**, 734.

STEELE, R.J.C., EREMIN, O., BROWN, M. & HAWKINS, R.A. (1984). A high macrophage content in human breast cancer is not associated with favourable prognostic factors. *Br. J. Surg.*, **71**, 456.

TARGAN, S.R. (1987). The intestine as an immunologic organ. *Ann. Intern. Med.*, **106**, 853.

UNDERWOOD, J.C.E. (1974). Lymphoreticular infiltration in human tumours: prognostic and biological implications: a review. *Br. J. Cancer*, **30**, 538.

VOSE, B.M. & MOORE, M. (1985). Human tumour-infiltrating lymphocytes: a marker of host response. *Semin. Haematol.*, **22**, 27.

WALDMANN, T.A. (1986). The structure, function and expression of interleukin-2 receptors on normal and malignant lymphocytes. *Science*, **232**, 727.